# Editorial: Photodynamic Therapy (3rd Edition)

**DOI:** 10.3390/biomedicines13061441

**Published:** 2025-06-11

**Authors:** Stefano Bacci

**Affiliations:** Research Unit of Histology and Embryology, Department of Biology, University of Florence, Viale Pieraccini 6, 50139 Florence, Italy; stefano.bacci@unifi.it

Photodynamic therapy (PDT) was first demonstrated in 1903 by Von Tappeiner and Jesionek, combining light therapy with a photosensitizer and oxygen [1]. PDT is widely used in medical treatments for various human disorders, producing reactive oxygen species that destroy oncological tissue and pathogens [2].

Recent studies show that mild PDT can also have beneficial effects, such as wound healing, stress and pathogen resistance, and extended lifespan in animal models [3,4].

This editorial provides an overview of “Photodynamic Therapy (3rd Edition)”, published in Biomedicine, highlighting recent contributions and advancements in PDT.

Research Articles

Gawecki et al. assessed the morphological and functional impacts of PDT in individuals with chronic central serous chorioretinopathy (CSCR), a retinal disorder characterized by the localized serous detachment of the macula [5]. The results indicated that a morphological response, characterized by the full clearance of subretinal fluid, was attained in 76.29% of patients, while an enhancement in best corrected visual acuity was seen in 77.53% of cases. All spectral domain optical coherence tomography assessments showed a substantial decrease after photodynamic therapy. Patients exhibiting intraretinal abnormalities or macular neovascularization had modest enhancements after PDT. A multivariate study indicated superior morphological outcomes correlated with younger age and male gender, as well as enhanced visual improvements in individuals devoid of intraretinal defects [6].

Regarding sensitizers [7], new boron dipyrromethene (BODIPY) compounds with a benzoxadiazole substituent have been recognized as promising sensitizers for PDT in cancer treatment. Porolnik et al. investigated the photochemical characteristics and cytotoxic activities of BODIPY in both normoxic and hypoxic environments. The dyes were analyzed via mass spectrometry and many nuclear magnetic resonance methods. In vitro investigations were performed on human ovarian cancer (A2780) and human breast adenocarcinoma (MDA-MB-231) cell lines. The incorporation of the benzoxadiazole moiety minimally influenced the location of the absorption maxima, but led to fluorescence quenching in comparison with mesophenyl-substituted analogs. Brominated and iodinated analogs exhibited significant light-induced cytotoxicity, characterized by low IC50 values ranging from 3.5 to 10.3 nM. Both compounds exhibited phototoxic action under hypoxic circumstances, with the iodinated BODIPY analog displaying the most significant cytotoxic impact. The research elucidates the benefits and possible limitations of BODIPY compounds, including heavy atoms and a benzoxadiazole moiety, as a valuable framework in medicinal chemistry for the development of novel photosensitizers [8].

The study of morphological changes in the skin during aging is a very current topic, and numerous research projects are being carried out. In general, with advancing age, collagen fibers diminish in thickness and transform in appearance, whilst elastic fibers exhibit fragmentation and a decrease in abundance. In the deep dermis, they gradually thicken due to distinct elastolysis mechanisms. The degradation and lysis of elastic fibers occur at a more rapid pace than in collagen fibers. With advancing age, the dermal vasculature diminishes owing to a decrease in both the quantity and size of vascular vessels, along with changes in the vascular wall constituents. These alterations progress until the vessel’s operation terminates [9,10,11,12]. In this research, Notari et al. used a 675 nm laser wavelength on cultured human dermal fibroblasts to elucidate the signaling pathways involved in skin regeneration. The fibroblasts were irradiated 24 h post seeding, followed by the application of immunofluorescence microscopy and Western blotting. The findings indicate that laser therapy elicits substantial alterations in fibroblasts, influencing the cytoskeletal structure and the synthesis and reorganization of extracellular matrix (ECM) components. The cellular response to the therapy mostly involves paxillin-mediated signaling pathways. These modifications indicate that laser therapy may enhance the architecture and functionality of dermal tissue, with ramifications for addressing skin aging [13].

Microviscosity is an essential metric of the internal resistance to flow in a fluid, exemplified by the lipid bilayer of a cell membrane. It affects systems such as metabolism, signaling, and cellular motility [14,15,16]. Nonetheless, alterations in this biophysical parameter during PDT remain inadequately understood [14,15,16]. In this work, Shimolina et al. investigated alterations in the microviscosity of live HeLa Kyoto tumor cells under photodynamic therapy with KillerRed, a genetically encoded photosensitizer, within several cellular localizations. The results show that the nuclear localization of KillerRed resulted in a progressive reduction in microviscosity, while its membrane localization initially rose in the first minutes of PDT before subsequently declining. The findings indicate that membrane microviscosity plays a role in tumor cell responsiveness to photodynamic therapy, contingent upon the location of reactive oxygen species targeting a genetically encoded photosensitizer [17].

Cervical cancer ranks as the fourth most common malignancy among women, with approximately 600,000 new cases and 340,000 related deaths occurring globally each year [18]. Persistent infection with high-risk human papillomavirus is strongly associated with the development of cervical cancer. Wei et al. examined the effectiveness of minimally invasive 5-aminolevulinic acid (ALA) PDT in the treatment of cervical high-grade squamous intraepithelial lesions (HSIL/CIN2). The results indicated that smoking and sleep disturbances were independent risk factors for unsuccessful lesion regression during ALA-PDT. This indicates the need for the meticulous evaluation of ALA-PDT for individuals with these disorders [19].

Glioblastoma, a rare malignant brain tumor, has a poor prognosis, with a median overall survival of 14.6 months and a 5-year survival rate of 9.8%, despite standard treatments like maximal surgical resection, 60-Gy radiotherapy, and chemotherapy [20,21,22].

Saito et al. investigated the efficacy of interstitial photodynamic treatment (i-PDT) for malignant gliomas in the brain. C6 glioma cells were introduced into the basal ganglia of rats, followed by intraperitoneal administration of the second-generation photosensitizer, talaporfin sodium (TPS). A prototype optical cable was inserted into the tumor tissue, and semiconductor laser light was emitted into the tumor under different settings. The brain was excised 24 h post i-PDT and subjected to pathological analysis. Histological investigation revealed that tumor necrosis occurred proximate to the light source, but that apoptosis was elicited at a distance. Elevated energy levels led to tissue edema, and irradiation at 75 J/cm^2^ was optimal for inducing apoptosis. The research developed an experimental i-PDT system using TPS, showing that tumor cell mortality is associated with light propagation [23].

Oral squamous-cell carcinoma and pancreatic cancer are lethal conditions that are becoming more prevalent and have low survival rates. In 2021, these two malignancies were responsible for 62,210 and 54,010 new cases, respectively [24,25]. The impact of a novel gel containing 5% *v*/*v* 5-ALA (ALAD-PDT) on human oral CAL-27 and pancreatic CAPAN-2 cancer cell lines was investigated in a study by D’Antonio et al. [26]. Flow cytometry demonstrated that the viability was greatly diminished at all concentrations, and that ALA-PDT induced significant apoptosis rates in both cancer cells. Both cell lines exhibited elevated levels of PpIX and ROS production. ALA-PDT has the potential to be administered as either a topical or intralesional therapy, which would enable the administration of modest dosages and reduce the likelihood of adverse effects. It has the potential to be a significant factor in the development of complex oral and pancreatic anticancer therapies [26].

Review

Bacterial infections and cancer are significant public health concerns on a global scale [27,28,29]. Antibiotics and corticosteroids are employed to treat bacterial infections, while chemotherapy medications, surgery, and radiotherapy are employed to combat cancer [27,28,29]. Resveratrol, a natural substance derived from Chinese herbal plants, has been employed to treat cancer and bacterial infections. It can also function as an adjuvant or photosensitizer in PDT. Resveratrol has been demonstrated to possess antibacterial and anticancer properties in studies that assess the efficacy of PDT against cancer and bacteria. Generally, it is used with hydrogels to improve the efficacy of PDT [30]. However, Law et al. conclude that additional research is required to evaluate the safety and cytotoxicity and improve the efficacy of PDT in various environments [31].

Discussion

In the context of this topic, there was an opportunity to appreciate both the clarity of the reviews and the proposed research articles, which include innovative techniques and treatment targets that will undoubtedly be further developed in the future. Based on the results of these studies, it can be concluded that PDT can generate growing optimism regarding the elimination of some diseases that have consistently plagued humanity.
